# Efficacy and safety comparison of hetrombopag and eltrombopag in children with cancer therapy-induced thrombocytopenia: a single-center retrospective study

**DOI:** 10.3389/fmed.2026.1890229

**Published:** 2026-08-20

**Authors:** Li-Ting Yu, Min Zhang, Jia-Xin Zhang, An-An Zhang, Zhuo Wang, Dan He, Hao-Nan Li, Zhi-Ling Li, Yi-Jin Gao

**Affiliations:** 1 Department of Clinical Pharmacy, Shanghai Children’s Medical Center, Shanghai Jiao Tong University School of Medicine, Shanghai, China; 2 Department of Pharmacy, Shandong Provincial Maternal and Child Health Care Hospital Affiliated to Qingdao University, Jinan, China; 3 Department of Hematology/Oncology, Shanghai Children’s Medical Center, Shanghai Jiao Tong University School of Medicine, Shanghai, China; 4 Department of Pharmacy, Hainan Branch, Shanghai Children’s Medical Center, School of Medicine, Shanghai Jiao Tong University, Sanya, China

**Keywords:** cancer treatment-induced thrombocytopenia, efficacy, eltrombopag, hetrombopag, safety

## Abstract

**Objective:**

To compare the efficacy and safety of hetrombopag and eltrombopag in children with cancer therapy-induced thrombocytopenia (CTIT), and to explore the clinical value of eltrombopag for this specific pediatric population given the current lack of dedicated literature.

**Methods:**

This was a single-center retrospective study. A total of 185 pediatric CTIT patients were enrolled, including 114 cases in the hetrombopag group and 71 cases in the eltrombopag group. Baseline characteristics, platelet (PLT) parameters, efficacy outcomes (platelet response rate, response duration, transfusion dependence), and adverse events (AEs) were compared between the two groups. Statistical analyses were performed using appropriate tests (t-test, Mann–Whitney U test, χ^2^ test, and proportional odds logistic regression), with *p* < 0.05 considered statistically significant.

**Results:**

Baseline characteristics were generally balanced between the two groups, with no significant differences in age, height, weight, primary diagnosis, or baseline PLT count (all *p* > 0.05), except for gender distribution (*p* = 0.013). Regarding efficacy: (1) Eltrombopag was associated with higher rates of partial or complete platelet response relative to hetrombopag in this retrospective cohort, with an odds ratio (OR) of 1.978 (95% confidence interval [95% CI]: 1.295 ~ 3.021, *p* = 0.001) after adjusting for confounding factors. (2) The rate of platelet transfusion dependence was significantly lower in the eltrombopag group (15.7% vs. 31.3%, *p* = 0.019). (3) No significant difference was observed in PLT count at drug withdrawal (*p* = 0.493) or response duration (39.42 ± 102.99 days vs. 63.75 ± 129.33 days, *p* = 0.192) between the two groups. No significant intergroup differences in response duration were found between hetrombopag and eltrombopag across the primary and three sensitivity analyses (all *p* > 0.05). For safety: (1) The hetrombopag group had higher incidences of Grade 1–2 leukopenia (41.2% vs. 23.9%, *p* = 0.016) and anemia (45.6% vs. 25.4%, *p* = 0.006). (2) The eltrombopag group had a higher incidence of elevated bilirubin levels (69.0% vs. 47.4%, *p* = 0.028). Thrombocytosis and thrombotic events were rare in both groups with no significant differences (*p* > 0.05).

**Conclusion:**

In pediatric CTIT patients, eltrombopag was linked to improved platelet response and less transfusion dependence, accompanied by a tolerable safety profile. As the first study focusing on eltrombopag for pediatric CTIT, these findings provide critical evidence supporting its clinical application as a promising therapeutic option for this understudied population.

## 1 Introduction

CTIT refers to thrombocytopenia in tumor patients caused by anti-tumor therapies during the course of disease treatment. It includes the clinically common chemotherapy-induced thrombocytopenia (CIT) reported previously, as well as thrombocytopenia induced by radiotherapy, targeted therapy, and immunotherapy (1). CTIT is one of the most common hematological toxicities during the treatment of pediatric malignancies, and it is particularly prominent in the chemotherapy-dominated comprehensive treatment modality (2). The treatment of pediatric malignancies (e.g., acute lymphoblastic leukemia, neuroblastoma, hepatoblastoma, etc.) is centered on high-intensity chemotherapy. Thrombocytopenia is not only a direct consequence of myelosuppression caused by chemotherapeutic agents (e.g., anthracyclines, platinum-based drugs, gemcitabine, etc.) (3), but may also be further exacerbated by factors such as radiotherapy, hematopoietic stem cell transplantation conditioning regimens, or infections (4). Thrombocytopenia not only elevates the risk of bleeding (e.g., petechiae, epistaxis, gastrointestinal bleeding, and even intracranial hemorrhage), but also directly limits the dose intensity and treatment continuity of chemotherapy. When the platelet count is <75 × 10^9^/L with bleeding tendency, chemotherapy must be delayed or platelet transfusion support should be administered (5). However, repeated transfusions may induce alloimmunization and platelet transfusion refractoriness (6), ultimately compromising the efficacy of tumor treatment and the long-term survival rate of pediatric patients.

In recent years, with the deepening understanding of the pathogenesis of CTIT and the development of novel thrombopoiesis-stimulating agents, remarkable progress has been made in its therapeutic strategies, ranging from traditional platelet transfusion and recombinant human interleukin-11 (rhIL-11) to recombinant human thrombopoietin (rhTPO) and next-generation thrombopoietin receptor agonists (TPO-RAs).

Although platelet transfusion, as a conventional supportive measure, can rapidly elevate platelet counts in emergency situations, it has limitations such as transient efficacy, risks of transfusion reactions, alloimmunization and blood-borne diseases. Repeated transfusions may even lead to platelet transfusion refractoriness, which seriously impairs the treatment process and long-term prognosis of pediatric tumor patients (7).

The advent of TPO-RAs has provided a targeted and sustainable solution for the management of CTIT. To date, TPO-RAs approved for marketing at home and abroad include romiplostim, eltrombopag, avatrombopag, hetrombopag and lusutrombopag, etc (8). Real-world cohort data demonstrated that weekly subcutaneous romiplostim-N01 achieved a higher 7-day marked platelet response rate and shorter chemotherapy delay time compared with daily rhIL-11/rhTPO for solid tumor-associated CTIT (9). In patients with acute myeloid leukemia complicated by CTIT, combined oral avatrombopag and rhIL-11 significantly accelerated platelet recovery without increasing adverse reaction risks relative to rhIL-11 monotherapy (10). Hetrombopag is a novel oral small-molecule non-peptide TPO-RA by selectively binding to and activating the thrombopoietin receptor (c-Mpl), hetrombopag promotes the proliferation, differentiation of megakaryocytes and subsequent platelet production. Different from natural TPO, its structurally optimized design avoids competitive binding with endogenous TPO, thereby reducing the risk of neutralizing antibody formation. Meanwhile, as a small-molecule agent, it features high oral bioavailability and does not require injection administration, which significantly improves the treatment compliance of pediatric patients (11).

The treatment of pediatric CTIT needs to take into account the characteristics of growth and development as well as drug safety. Chemotherapy regimens for pediatric malignancies are typically more intensive, making them more prone to inducing clinically significant cytopenia. Studies have shown that the incidence of CTIT in pediatric tumor patients is 20–30% higher than that in adults (2). Meanwhile, there are significant age-related differences in platelet counts among children: the lower limit of normal platelet count can reach 150 × 10^9^/L in infants and young children (1–3 years old), which is close to that of adolescents and adults. The clinical application experience of TPO-RAs in children is limited. rhTPO- class agents have not been approved for the treatment of CTIT in pediatric patients. Eltrombopag has been approved by the FDA for the treatment of immune thrombocytopenia (ITP) in patients aged ≥1 year, with the recommended dosage adjusted according to body weight and age. Although some studies have attempted to extend the use of adult TPO-RAs (e.g., eltrombopag) to pediatric populations, clinical application remains cautious due to unclear pediatric dosage regimens, undefined pharmacokinetic characteristics, and insufficient long-term safety data. Based on this, this study aims to explore the real-world differences in efficacy and safety between hetrombopag and eltrombopag in the treatment of pediatric CTIT patients. It holds great clinical significance for filling the gap in targeted therapy for pediatric CTIT, reducing platelet transfusion dependence, and ensuring the continuity of chemotherapy.

## 2 Methods

### 2.1 Ethics

This retrospective single-center clinical study was conducted at Shanghai Children’s Medical Center, Shanghai, China. The study was conducted in accordance with the Declaration of Helsinki and its subsequent amendments. The study was approved by the Institutional Review Board of Shanghai Children’s Medical Center, School of Medicine, Shanghai Jiao Tong University (No. SCMCIRB-K2025021-1). Individual consent for this retrospective analysis was waived.

### 2.2 Participants

This single-center, retrospective cohort study analyzed children diagnosed with CTIT at Shanghai Children’s Medical Center. We retrospectively enrolled all adult patients receiving either hetrombopag or eltrombopag between January 2024 and July 2025 from our institutional electronic medical records. Treatment assignment was determined by attending clinicians based on individual patient conditions, laboratory parameters and clinical judgment rather than formal randomization. Group sizes differed due to unequal real-world prescription frequency of the two thrombopoietin receptor agonists during the study period. All eligible patients with evaluable duration of platelet response data were retained for analyses. As an exploratory, hypothesis-generating analysis, no formal sample size calculation was performed. Participants were divided into the hetrombopag group and the eltrombopag group according to the actual medication regimens they received.

### 2.3 Inclusion criteria

(1) Aged 1 year ≤ age < 17 years, regardless of gender; (2) Clinically diagnosed with CTIT; (3) Expected survival time of ≥ 12 weeks; (4) No administration of TPO-RA agents (e.g., hetrombopag, eltrombopag, avatrombopag, romiplostim) within one week.

### 2.4 Exclusion criteria

(1) Patients with uncontrollable bleeding and/or infection despite standard treatment prior to enrollment; (2) Patients with other hematopoietic system diseases apart from chemotherapy-induced thrombocytopenia; (3) Patients with significant hepatic or renal dysfunction; (4) Patients with a history of liver cirrhosis or portal hypertension; (5) Patients who have experienced drug-requiring congestive heart failure, arrhythmia, or peripheral arteriovenous thrombosis within 1 year prior to enrollment, or myocardial infarction or cerebral infarction within 3 months prior to enrollment.

### 2.5 Research methods

Patients in the eltrombopag group were administered eltrombopag olamine tablets (Novartis Pharma Schweiz AG, specification: 25 mg per tablet) for oral administration. Patients in the hetrombopag group were given hetrombopag olamine tablets (Jiangsu Hengrui Pharmaceuticals Co., Ltd., specification: 2.5 mg per tablet) for oral administration.

#### 2.5.1 Dosing regimens

1. Eltrombopag: 1–5 y, 25 mg QD; 6–11 y ≥ 27 kg: 50 mg QD; <27 kg: 25 mg QD; 12–17 y, 50 mg QD, with the maximum dose not exceeding 75 mg per day.
2. Hetrombopag: 1–5 y, 2.5 mg QD; 6–11 y, 5 mg QD; 12–17 y, 7.5 mg QD.

The treatment continued until the platelet count exceeded 50 × 10^9^/L for 7 consecutive days and platelet transfusion was no longer required.

### 2.6 Efficacy evaluation

(1) The platelet (PLT) count at the time of treatment discontinuation was significantly increased compared with the baseline. After treatment, complete response (CR) was defined as a stable PLT count ≥ 50 × 10^9^/L for at least one week without bleeding; partial response (PR) was defined as a PLT count reaching 20–50 × 10^9^/L (at least twice the baseline level) sustained for at least one week without bleeding; no response (NR) was defined as a PLT count < 20 × 10^9^/L or persistent dependence on platelet transfusion even after treatment with the maximum tolerated dose (2) Reduction in the platelet transfusion rate. (3) Prolongation of the duration of platelet response (days with PLT > 50 × 10^9^/L). The definitions of CR, PR, and NR for platelet count were based on the guidelines from the American Society of Clinical Oncology (ASCO) and the Children’s Oncology Group (COG), as well as core clinical studies on thrombopoietin receptor agonists (TPO-RAs) in pediatric patients (12, 13).

### 2.7 Adverse reaction assessment

AEs were actively identified by trained study personnel through a comprehensive review of electronic medical records, including laboratory data, physician and nursing notes, medication records. Each identified AE was graded according to CTCAE v4.0 (14) and categorized based on WHO terminology.

### 2.8 Statistical analysis

SPSS 29.0 (IBM Corp., Armonk, New York, USA) was used for analysis. Descriptive statistics were used to summarize patient demographics and clinical characteristics. Continuous data were first evaluated for normality using the Shapiro–Wilk test. If they followed a normal distribution (*p* > 0.05), they were expressed as mean ± standard deviation, and comparisons were performed using the independent samples t-test. If they did not conform to a normal distribution (*p* < 0.05), they were presented as median (interquartile range) [M (P25, P75)], and comparisons were conducted using the Mann–Whitney U test. Categorical data were expressed as the number of cases and percentage [*n* (%)]. Paired samples t-test was used for comparing relevant indicators before and after treatment within the same group, and inter-group comparisons were performed using the chi-square test or Fisher’s exact probability test. A *p* value < 0.05 was considered statistically significant.

## 3 Results

### 3.1 Baseline characteristics

**Figure 1:**
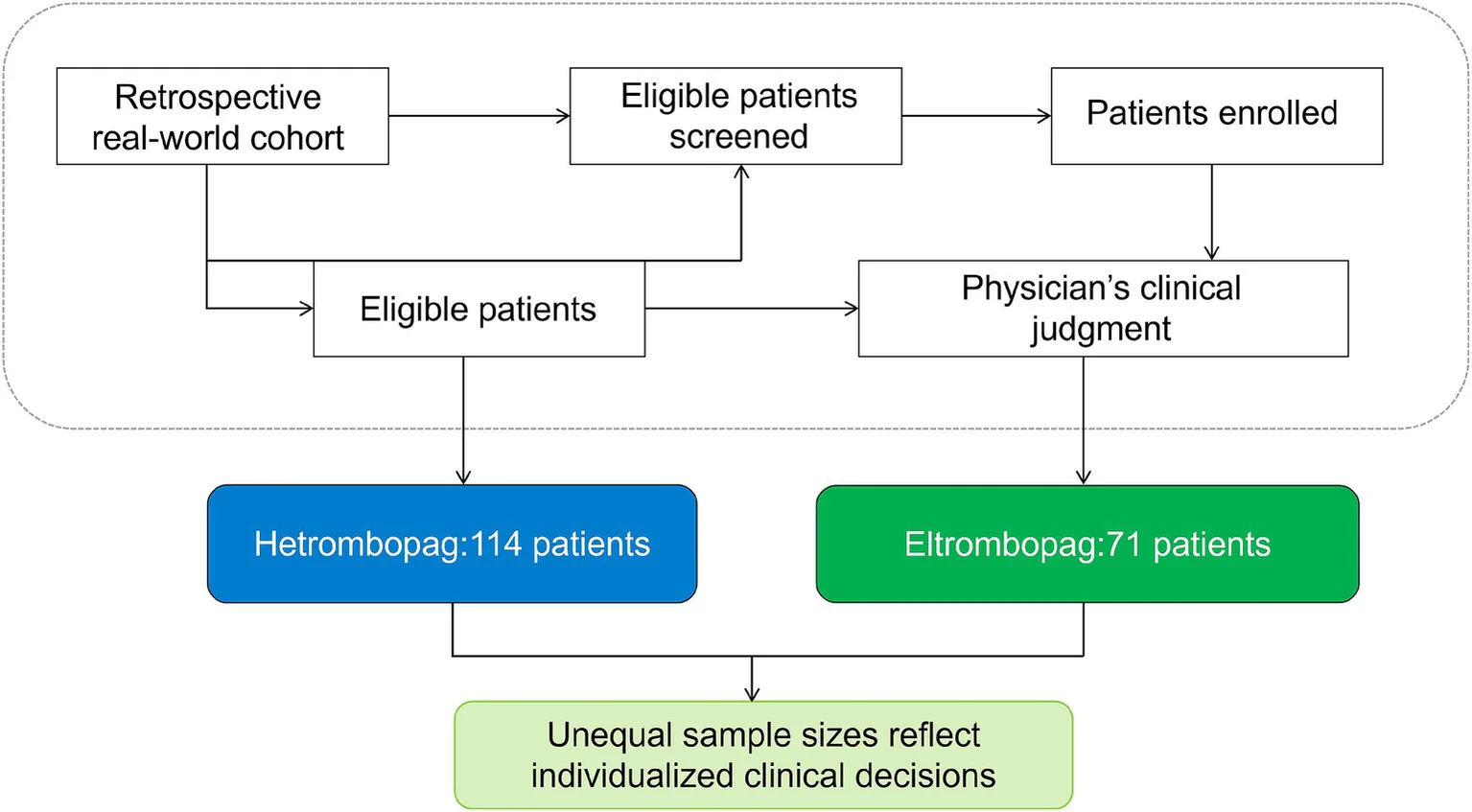
Patient selection flow diagram.

A total of 185 patients were enrolled in this study, including 114 cases in the hetrombopag group and 71 cases in the eltrombopag group. In the hetrombopag group, there were 80 males and 34 females; in the eltrombopag group, there were 37 males and 34 females. The patient screening process is presented in Figure 1.

Comparison of the general data including age, height, body weight diagnosis and Chemotherapy cycles between the hetrombopag group and the eltrombopag group showed no statistically significant difference (*p* > 0.05), while there was a statistically significant difference in gender distribution between the two groups (*p* < 0.05) (Table 1). To eliminate confounding from this disparity and other prognostic factors, all analyses of duration of response (DoR) across four sensitivity scenarios adopted an identical fully adjusted multivariable linear regression model incorporating all predefined covariates: gender, age, baseline platelet count, disease duration, and prior immunosuppressive therapy. Only adjusted between-group differences from the full model are presented in all main tables.

**Table 1:** Baseline characteristics of patients.

Characteristics	Hetrombopag group (*n* = 114)	Eltrombopag group (*n* = 71)	Z/χ^2^	*p* value
Gender (male) [*n*%]	80 (70.17)	37 (52.11)	2.51	0.013
Age (years, $\bar{x}$ ± s)	9.67 ± 4.57	9.02 ± 4.15	0.971	0.333
Height (cm, $\bar{x}$ ± s)	133.53 ± 29.82	131.03 ± 27.12	0.575	0.566
Weight (kg, $\bar{x}$ ± s)	33.46 ± 17.93	31.38 ± 15.35	0.807	0.421
Diagnosis			0.243	0.998
Acute Lymphoblastic Leukemia	45	31	0.018	0.893
Acute Myeloid Leukemia	23	13	0.132	0.716
Burkitt Lymphoma	25	17	0.012	0.913
Hepatoblastoma	5	2	0.004	0.949
T-cell Lymphoproliferative Disorder	7	5	0.007	0.934
Rhabdomyosarcoma	3	1	0.041	0.840
Others	6	2	0.021	0.885
Chemotherapy cycles			0.018	>0.05
Induction chemo	44	47		
Isolated HSCT	33	10		
CAR-T related	22	14		
Others	15	7		

Post-hoc power analysis for the primary endpoint demonstrated a power of 28.14% at α = 0.05 two-sided, indicating the study was underpowered to detect small-magnitude between-group differences.

### 3.2 Comparison of platelet counts

**Figure 2:**
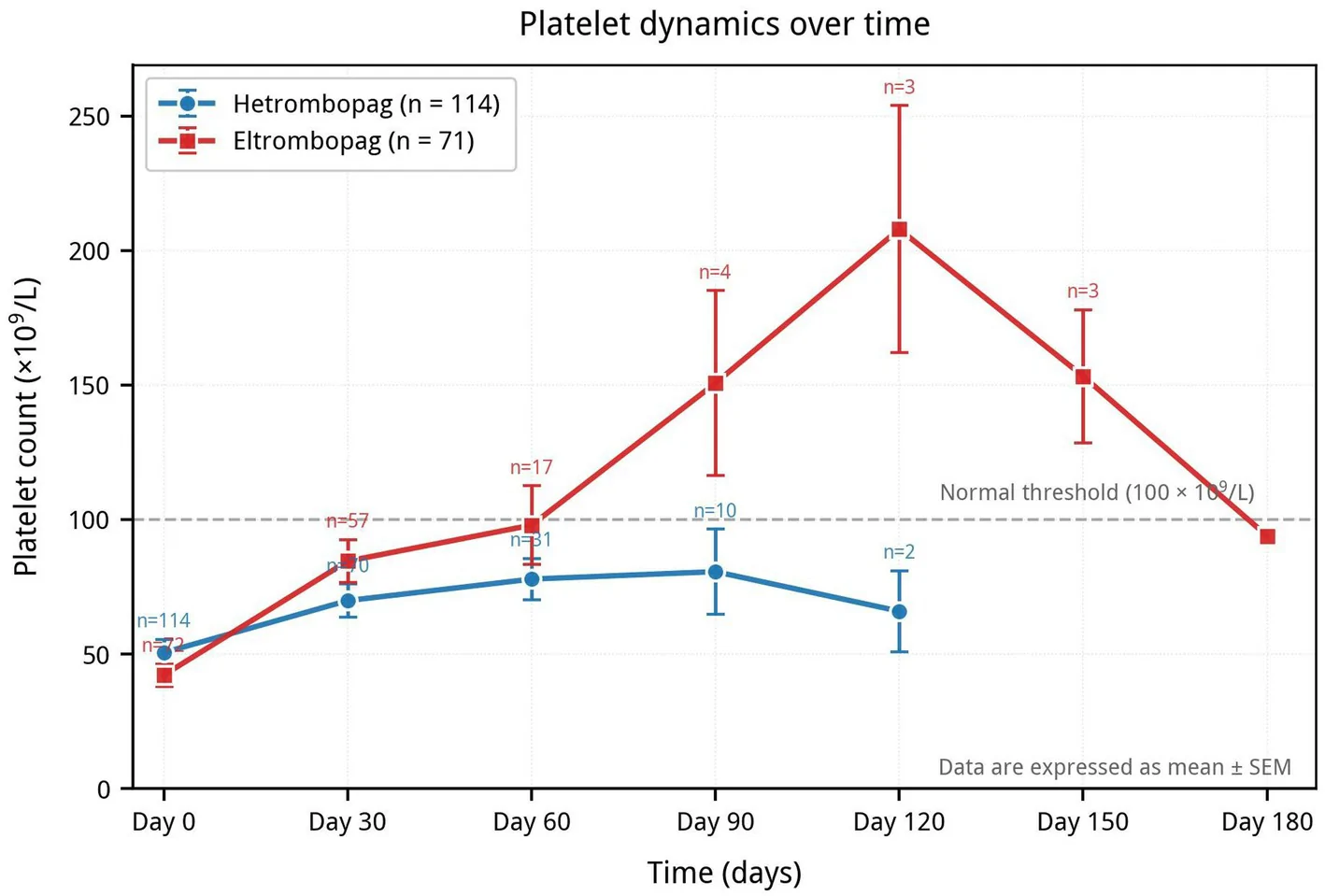
Platelet dynamics over time.

Longitudinal changes in platelet counts are illustrated in Figure 2. The platelet levels of the two patient groups were comparable before treatment (*p* = 0.337). Compared with the baseline, the platelet counts of both groups increased significantly at the time of drug withdrawal after treatment, while there was no statistically significant difference in the platelet levels between the two groups at the time of drug withdrawal (*p* = 0.493), as shown in Table 2.

**Table 2:** Comparison of PLT parameters.

PLT	Hetrombopag group (*n* = 114)	Eltrombopag group (*n* = 71)	Z/χ^2^	*p* value
Baseline PLT (×10^9^/L)	46.93 ± 50.35	40.44 ± 33.17	0.963	0.337
PLT at drug withdrawal (×10^9^/L)	71.96 ± 81.90	79.81 ± 62.36	0.688	0.493
Duration of TPO-RA treatment	44.63 ± 23.67	35.68 ± 21.25	U = 402	0.139
Platelet transfusion dependence	35 (31.3)	11 (15.7)	5.505	0.019

### 3.3 Efficacy evaluation

#### 3.3.1 Comparison of platelet response rates between two groups

##### 3.3.1.1 Group effect

Compared with the hetrombopag group, the eltrombopag group had an odds ratio (OR) of 1.978 (95% confidence interval [95% CI]: 1.295 ~ 3.021) for achieving “a higher efficacy grade” (i.e., partial response [PR] or complete response [CR]), with a statistical significance of *p* = 0.001 < 0.05. This fully adjusted analysis demonstrated that eltrombopag treatment was significantly associated with higher platelet response rates relative to hetrombopag.

##### 3.3.1.2 Model validity

The proportional odds assumption was verified by the Wald test (Wald χ^2^ = 3.25, *p* = 0.197 > 0.05), which confirmed the validity of the assumption and indicated a good model fit. As shown in Table 3.

**Table 3:** Comparison of platelet response rates.

Variable	Regression coefficient (β)	Standard error (SE)	Wald χ^2^	*p* value	Odds ratio (OR)	95% confidence interval (95%CI)
Grouping (Eltrombopag vs. Hetrombopag)	0.682	0.215	10.12	0.001	1.978	1.295 ~ 3.021
Intercept 1 (Y ≥ 1 VS Y < 1)	−0.854	0.189	20.56	<0.001	-	-
Intercept 2 (Y ≥ 2 vs. Y < 2)	0.326	0.192	2.92	0.087	-	-

#### 3.3.2 Sensitivity analysis of platelet response duration

The comparable platelet response durations of hetrombopag and eltrombopag in pediatric CTIT patients were highly robust across all clinically relevant analyses, as shown in Table 4. Across all four analytical scenarios (primary analysis and three sensitivity analyses), no statistically significant between-group difference in the duration of treatment response was detected between hetrombopag and eltrombopag, with all *p*-values greater than 0.05. Consistent trends of comparable mean and median response durations under varied exclusion/inclusion criteria confirm the robustness of the primary finding that the two thrombopoietin receptor agonists yield similar response persistence in eligible patients, as shown in Table 5.

**Table 4:** Comparison of platelet response duration.

Platelet response duration	Hetrombopag group (*n* = 114)	Eltrombopag group (*n* = 71)	Z/χ^2^	*p* value
Duration of response (PR, days)	39.42 ± 102.99	63.75 ± 129.33	1.311	0.192

**Table 5:** Sensitivity analysis results of duration of response in two groups of patients.

Analysis scenario	Treatment group	*n*	Mean ± SD (days)	Median (IQR, days)	Range (min-max, days)	*p*-value
Primary analysis (all responders)	Hetrombopag	73	118.99 ± 153.39	32.00 (7.00, 190.00)	2.00–524.00	-
	Eltrombopag	33	129.42 ± 160.41	58.00 (9.00, 190.00)	2.00–524.00	0.754
Sensitivity analysis 1: Excluding responses <7 days	Hetrombopag	60	144.00 ± 158.55	78.50 (12.75, 262.25)	7.00–524.00	-
	Eltrombopag	28	152.00 ± 164.34	95.00 (13.50, 267.75)	7.00–524.00	0.831
Sensitivity analysis 2: Excluding 5% extreme values	Hetrombopag	69	96.94 ± 126.09	23.00 (7.00, 141.00)	2.00–475.00	-
	Eltrombopag	31	104.94 ± 131.17	32.00 (8.50, 152.50)	2.00–475.00	0.777
Sensitivity analysis 3: Only including follow-up ≥30 days	Hetrombopag	37	226.05 ± 152.07	190.00 (95.00, 314.00)	32.00–524.00	-
	Eltrombopag	18	230.00 ± 157.63	177.00 (111.25, 337.25)	32.00–524.00	0.930

### 3.4 Adverse reactions evaluation

The incidence of hematological adverse reactions was significantly higher in the hetrombopag group, including anemia and leukopenia (Grade1-2), with statistically significant differences (*p* < 0.05). By contrast, the incidence of hepatic dysfunction was higher in the eltrombopag group, which was mainly manifested as elevated bilirubin, and the difference was statistically significant (*p* < 0.05), as shown in Table 6.

**Table 6:** AEs reported among participants.

AEs	Hetrombopag group (*n* = 114)	Eltrombopag group (*n* = 71)	Z/χ^2^	*p* value
Leukopenia (Grade1-2)	47 (41.2)	17 (23.9)	5.777	0.016*
Leukopenia (Grade3-4)	40 (35.1)	16 (22.5)	3.266	0.071
Anemia (<65 g/L)	52 (45.6)	18 (25.4)	7.637	0.006*
Increased Bilirubin Levels (>17.1 μmol/L)	54 (47.4)	49 (69.0)	4.851	0.028*
Increased Serum Creatinine (>88 umol/L)	39 (34.2)	23 (32.4)	0.065	0.799
Thrombocytosis (PLT>400 × 10^9^/L)	1 (0.9)	0 (0)	0.643	0.498
Thrombotic event	2 (1.7)	0 (0)	1.268	0.308

AE, adverse event. *p < 0.05.

## 4 Discussion

CTIT remains a critical and common adverse event in pediatric oncology, often leading to chemotherapy dose delays, dose reductions, or even treatment discontinuation, which significantly compromises therapeutic outcomes and increases the risk of life-threatening bleeding (15). Thrombopoietin receptor agonists (TPO-RAs) have emerged as a promising therapeutic option for CTIT by stimulating platelet production, but data on their efficacy and safety in pediatric populations, particularly head-to-head comparisons between different TPO-RAs, are extremely limited. To our knowledge, this is the first retrospective study to directly compare the clinical value of hetrombopag and eltrombopag in children with CTIT, filling the gap in current evidence where no dedicated literature on eltrombopag for pediatric CTIT exists.

Efficacy of Eltrombopag in Pediatric CTIT The baseline characteristics of the two groups were generally balanced, with no significant differences in age, height, weight, or primary cancer diagnoses, ensuring the comparability of efficacy outcomes. Notably, despite a statistically significant difference in gender distribution (more males in the hetrombopag group), this confounding factor was accounted for in the efficacy analysis. This study’s primary adjusted finding showed eltrombopag correlated with greater likelihood of PR or CR versus hetrombopag, with an odds ratio (OR) of 1.978 (95% CI: 1.295 ~ 3.021, *p* = 0.001). This indicates that pediatric patients with CTIT treated with eltrombopag are nearly twice as likely to achieve meaningful platelet recovery as those treated with hetrombopag. The apparent link between eltrombopag and improved platelet response merits attention, given scarce prior evidence for its use in pediatric CTIT. While TPO-RAs have been approved for immune thrombocytopenia (ITP) in children (16), their application in CTIT is off-label, and data specific to this population are scarce. A previous study by Liu P et al. demonstrated the efficacy of eltrombopag in adult CTIT, with a significant increase in platelet counts and reduced transfusion requirements (17), but extrapolating adult data to children is inappropriate due to differences in pharmacokinetics, pharmacodynamics, and cancer treatment regimens. This retrospective cohort detects a correlation between eltrombopag and improved platelet response rates in pediatric CTIT. While these observational data imply eltrombopag could serve as a promising first-line TPO-RA choice, no definitive causal or comparative efficacy claims can be established. Additionally, while there was no significant difference in platelet response duration between the two groups (*p* = 0.192), the mean response duration in the eltrombopag group (63.75 ± 129.33 days) was numerically longer than that in the hetrombopag group (39.42 ± 102.99 days), suggesting a potential sustained therapeutic effect. The lack of statistical significance may be attributed to the wide variability in response duration, which could be influenced by factors such as underlying cancer type, treatment intensity, and individual patient characteristics. Furthermore, eltrombopag was associated with a significantly lower rate of platelet transfusion dependence (15.7% vs. 31.3%, *p* = 0.019), a key clinical endpoint in CTIT management. Reduced transfusion dependence not only alleviates the burden of blood product resources but also minimizes transfusion-related risks such as infection, alloimmunization, and transfusion reactions (18), which are particularly relevant in pediatric patients with immature immune systems.

Safety Profile of Eltrombopag in Pediatric CTIT Safety is a paramount concern in pediatric oncology, and our study revealed distinct adverse event (AE) profiles between the two TPO-RAs. Eltrombopag was associated with a higher incidence of elevated bilirubin levels (69.0% vs. 47.4%, *p* = 0.028), which was the most notable safety difference. However, it is important to note that this elevation was primarily mild to moderate and reversible, consistent with previous reports of eltrombopag-related hepatobiliary AEs in adult and pediatric ITP patients (19). The mechanism underlying bilirubin elevation may be related to eltrombopag’s metabolism in the liver, but the clinical significance is limited as no patients developed severe liver dysfunction or required treatment discontinuation. Liver function monitoring is recommended for patients receiving eltrombopag, yet this adverse event does not warrant avoidance of the agent in view of its observed correlation with improved platelet responses. In contrast, the hetrombopag group had significantly higher incidences of hematological AEs, including Grade 1–2 leukopenia (41.2% vs. 23.9%, *p* = 0.016) and anemia (45.6% vs. 25.4%, *p* = 0.006). These findings are clinically relevant because leukopenia and anemia further compromise the already fragile hematopoietic function of pediatric cancer patients, increasing the risk of infection and fatigue. Other AEs, such as thrombocytosis (PLT > 400 × 10^9^/L) and thrombotic events, were rare in both groups with no significant differences, confirming the overall safety of both TPO-RAs in pediatric CTIT.

### 4.1 Limitations

The favorable platelet response correlates and acceptable safety findings for eltrombopag observed herein bear meaningful clinical relevance. Given the lack of dedicated pediatric CTIT guidelines and the paucity of comparative data, our findings provide valuable evidence for clinical decision-making. Eltrombopag should be considered a preferred TPO-RA for pediatric patients with CTIT, especially those at high risk of bleeding or transfusion dependence. However, individual patient factors such as baseline liver function should be taken into account, and liver function monitoring is essential during treatment. This study has several limitations that should be acknowledged. First, it is a single-center retrospective study with a relatively limited sample size (*n* = 185), which may introduce selection bias and limit the generalizability of the results. Second, the follow-up duration was not standardized, and long-term outcomes such as sustained platelet recovery and late AEs were not evaluated. Third, the study did not collect data on pharmacokinetic parameters or genetic factors that may influence treatment response and safety. Future prospective, multi-center studies with larger sample sizes and longer follow-up are needed to validate our findings and establish optimal dosing regimens for eltrombopag in pediatric CTIT.

## 5 Conclusion

This is the first study to compare hetrombopag and eltrombopag in children with CTIT, providing critical evidence for the use of eltrombopag in this understudied population. In this retrospective cohort, eltrombopag correlated with improved platelet responses and less transfusion reliance, with reversible bilirubin increases as its primary manageable adverse event. These findings support the clinical value of eltrombopag as a promising therapeutic option for pediatric CTIT, addressing the unmet need for evidence-based TPO-RA use in children with cancer. Further prospective studies are warranted to confirm these results and optimize the management of CTIT in pediatric oncology.

## Data Availability

The datasets presented in this article are not readily available because the relevant dataset shall not be disclosed or shared with external parties. Requests to access the datasets should be directed to yuliting20091396@163.com.
